# Race categorization in noise

**DOI:** 10.1177/20416695221119530

**Published:** 2022-08-30

**Authors:** Peter de Lissa, Katsumi Watanabe, Li Gu, Tatsunori Ishii, Koyo Nakamura, Taiki Kimura, Amane Sagasaki, Roberto Caldara

**Affiliations:** 27211 University of Fribourg, Fribourg, Switzerland; 13148 Waseda University, Tokyo, Japan; School of Innovation Design, 34741 Guangzhou Academy of Fine Arts, Guangzhou, China; Japan Womens' University, Tokyo, Japan; Waseda University, Tokyo, Japan; University of Vienna, Vienna, Austria; Japan Society for the Promotion of Science, Tokyo, Japan; Waseda University, Tokyo, Japan; 13148 Waseda University, Tokyo, Japan; 13148 Waseda University, Tokyo, Japan; 27211 University of Fribourg, Fribourg, Switzerland

**Keywords:** face processing, race, other-race face categorization advantage

## Abstract

People are typically faster to categorize the race of a face if it belongs to a race different from their own. This Other Race Categorization Advantage (ORCA) is thought to reflect an enhanced sensitivity to the visual race signals of other race faces, leading to faster response times. The current study investigated this sensitivity in a cross-cultural sample of Swiss and Japanese observers with a race categorization task using faces that had been parametrically degraded of visual structure, with normalized luminance and contrast. While Swiss observers exhibited an increasingly strong ORCA in both reaction time and accuracy as the face images were visually degraded up to 20% structural coherence, the Japanese observers manifested this pattern most distinctly when the faces were fully structurally-intact. Critically, for both observer groups, there was a clear accuracy effect at the 20% structural coherence level, indicating that the enhanced sensitivity to other race visual signals persists in significantly degraded stimuli. These results suggest that different cultural groups may rely on and extract distinct types of visual race signals during categorization, which may depend on the available visual information. Nevertheless, heavily degraded stimuli specifically favor the perception of other race faces, indicating that the visual system is tuned by experience and is sensitive to the detection of unfamiliar signals.

Our faces offer insight into who we are, presenting others with our identity and an idea of our age, gender, and emotions. Some of these visual properties are more nuanced than others, requiring greater experience and expertise to recognize. An example of this difference in the way faces can be processed and categorized can be found when we turn to the realm of how “race” is perceived in faces. It is common to find that when we lack sufficient experience with people of races with characteristic physiognomic features different than our owns, we find it more difficult to identify them. Termed the “Other-Race Effect” (ORE), this difference in identity recognition is associated with an asymmetry in the number of faces of differing races we encounter in our lives compared to those of our own race—the less familiar we are with other-race faces the greater the deficit in identity recognition we have with such faces (Brigham & Barkowitz, 1978; Fallshore & Schooler, 1995; Meissner & Brigham, 2001; Rhodes et al., 2009; Valentine & Endo, 1992). An interesting companion to this effect is that while we are better at recognizing members of our own (familiar) race, we are actually faster to categorize the race of a face when it belongs to other (less familiar) race (Caldara et al., 2004; Feng et al., 2011; Ge et al., 2009; Levin, 1996, 2000; Valentine & Endo, 1992; Zhao & Bentin, 2008, 2011). Referred to as the Other Race Categorization Advantage (ORCA), this effect has been proposed to stem from a difference in the amount and types of visual information we have encountered and stored through our experiences with faces throughout our lives. Valentine (1991), Valentine and Endo (1992) put forth the multidimensional face-space model to account for both the ORE and ORCA patterns, positing that the more limited number of other race exemplars (encountered faces) reflects a reduced space for recognition abilities relative to a more elaborated same-race exemplar area and that the smaller other race area is also more dense due to greater visual overlap. This theoretical model has been statistically validated by neuronal network simulations (Caldara & Abdi, 2006).Valentine (1991) suggested that the visual similarity of other-race faces creates both decreased recognition abilities as well as enhanced race categorization abilities, by relying on a sensitivity to the visual signals for a race at the expense of the more nuanced signals used for individuation. Levin (1996, 2000) further proposed that race can be seen as a distinct feature of faces that are emphasized in other race faces by virtue of it being a salient visual aspect used to categorize faces into social groups. The decreased recognition ability people tend to possess for other race faces was thus hypothesized to be a consequence of the emphasis placed on race as a feature in other race faces. Further support for the ORCA to stem from an enhanced sensitivity to the visual characteristics representing race has been presented in the form of diffusion model analysis by Benton and Skinner (2015), whereby the evidence accumulation rate in a two forced-choice race categorization task was systematically related to the observers’ race—Caucasian participants were more sensitive to “Asian” visual cues than Caucasian, and the reverse pattern was observed in the Asian participant sample. To tap into this enhanced sensitivity, Benton and Skinner used a series of faces that had been morphed to systematically control the levels of Asian and Caucasian race signals in each presented face, allowing for the rate of accumulation to be derived from the response times of the participants. Such an approach is particularly appropriate for the investigation of the ORCA, which is most consistently found to be indexed through reaction times rather than accuracy (Caldara et al., 2004; Ge et al., 2009; Liu et al., 2015; Valentine & Endo, 1992; Zhao & Bentin, 2008, 2011) although not exclusively (cf. Feng et al., 2011). However, if the ORCA is an index of how sensitive we are to the visual race signals of the same and other race faces, we may be able to probe this dynamic by systematically decreasing the strength of the input of these signals. From this point of view, one may expect there to be a tipping point at which the enhanced sensitivity to other race faces allows for preserved race categorization relative to same race faces, leading to accuracy differences at specific levels of visual input. To address this question, we presented participants with images of East Asian (EA) and West Caucasian (WC) faces and asked them to categorize the race of the face. Critically, we systematically scrambled the phase/location of the spatial frequencies in the images in incremental steps of 5%, ranging from 100% structure through to 0% structure (Honey et al., 2008; Kuefner et al., 2010; Rodger et al., 2015; Rodger et al., 2018; Rousselet et al., 2008; Stoll et al., 2019; Wyssen et al., 2019). Comparisons across this range of phase coherence allowed us to compare the accuracy and reaction time profiles of a group of Swiss and a group of Japanese participants to determine how race categorization is performed in visually degraded stimuli.

## Methods

The Human Ethics Committee at the University of Fribourg and the University of Waseda approved the methods and procedure used in this study. All participants provided written informed consent in accordance with the Declaration of Helsinki. All data used in statistical analyses and represented in figures are available online on the Open Science Framework repository at https://osf.io/s6gwj/.

### Participants

Participants were recruited from two separate cultural groups; 60 Swiss undergraduate students from the University of Fribourg (West Caucasian), and 66 Japanese participants from Waseda University (East Asian). After the dataset inclusion/exclusion process (see section “Data Processing & Analysis”), there were 38 participants in the Swiss participants sample (*M*_age_ = 20.29, *SD*_age_ = 3.38, 33 female, 36 right-handed), and 46 in the Japanese sample (*M*_age_ = 22.23, *SD*_age_ = 3.89, 24 female, 41 right-handed). Six participants in the Japanese sample declined to give their ages. All participants reported having normal or corrected-to-normal vision and were given research participation credit for their respective studies.

### Stimuli

**Figure 1.**
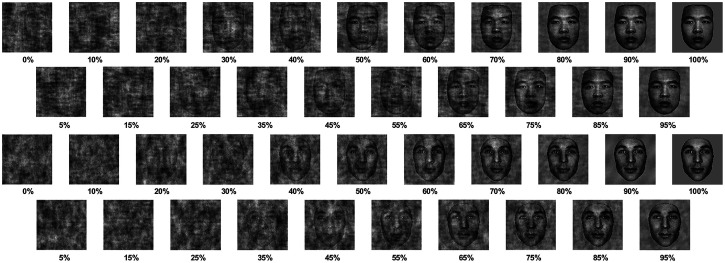
Examples of EA and WC face stimuli used in the study, ranging from 0% structural coherence through to 100%, in 5% steps. The WC and EA were matched on low-level visual qualities at a level of coherence.

Note. EA = East Asian; WC = West Caucasian.

The face stimuli consisted of 20 grayscale images of 10 Caucasian and 10 Asian identities (equal number of male and female). The face images were neutral expression portrait photographs of Belgian (WC) and Chinese (EA) students aged from 18 to 25 years, and have been utilized in previous studies investigating same and other race face processing (de Lissa et al., 2021; Michel et al., 2006, 2006). The faces were cropped to exclude ears and hair, and were matched for amplitude spectra, luminance, and contrast using the SHINE toolbox (Willenbockel et al., 2010). To systematically degrade the structure of the face images, we utilized a phase-scrambling technique that randomized the location of all spatial frequencies in the images while preserving amplitude spectra across orientations and spatial frequencies (Honey et al., 2008). We thus subjected the face images to this phase randomization process in 5% steps of preserved coherence that ranged from 100% (full coherence/image structure) to 0% (completely randomized, no image structure) for a total of 21 levels of phase coherence (see Figure 1). The phase-randomization process was performed twice to produce two sets of phase-scrambled stimuli to create a broader array of randomization patterns. There were 20 stimulus images in each experimental condition, with a total of 840 trials presented to participants. Prior to the commencement of the experiment, the participants calibrated the presentation of the stimuli size on their computer screens by scaling an image to match the size of a bank card and noting their distance from the screen. Accordingly, the face stimuli were presented to the dimensions of 9.78° × 13.29° of visual angle set within a square image subtending 15.1° on each side.

### Procedure

The stimuli were presented through web-based Gorilla Experiment Builder software (www.gorilla.sc) to create and host our experiment (Anwyl-Irvine et al., 2020, 2021), and conducted on personal desktop/laptop computers of participants in an environment of their choosing. These choices were a compromise due to lockdown situations in both countries imposed by COVID-19 restrictions. Participants were instructed to categorize faces according to their race, pressing either the “F” or “J” keys with separate hands, with the respective conditions counterbalanced between two groups of participants to avoid laterality effects. Participants were instructed to respond “as quickly and accurately as possible,” and task instructions were accordingly translated for Japanese participants. Four practice trials allowed participants to learn the race/key response instructions, with accuracy feedback provided after each trial to ensure the response hands were learned correctly. After the practice trials, the experimental trials commenced where no feedback was provided. Trials commenced with a central fixation cross presented for an average of 600 ± 100 ms (jittered), followed by a 200 ms blank screen before a face stimulus was presented for 200 ms before being replaced by a blank screen. The choice of a 200 ms face stimulus presentation duration was guided by the results of a previous study indicating that race categorization can be reliably executed within 200 ms of face presentation (de Lissa et al., 2021). The trial ended when a participant made a key response, which was proceeded by an 800 ± 200 ms (jittered) blank screen. Six breaks were provided throughout the testing sessions, which lasted approximately 45 min.

### Data Processing & Analysis

**Figure 2.**
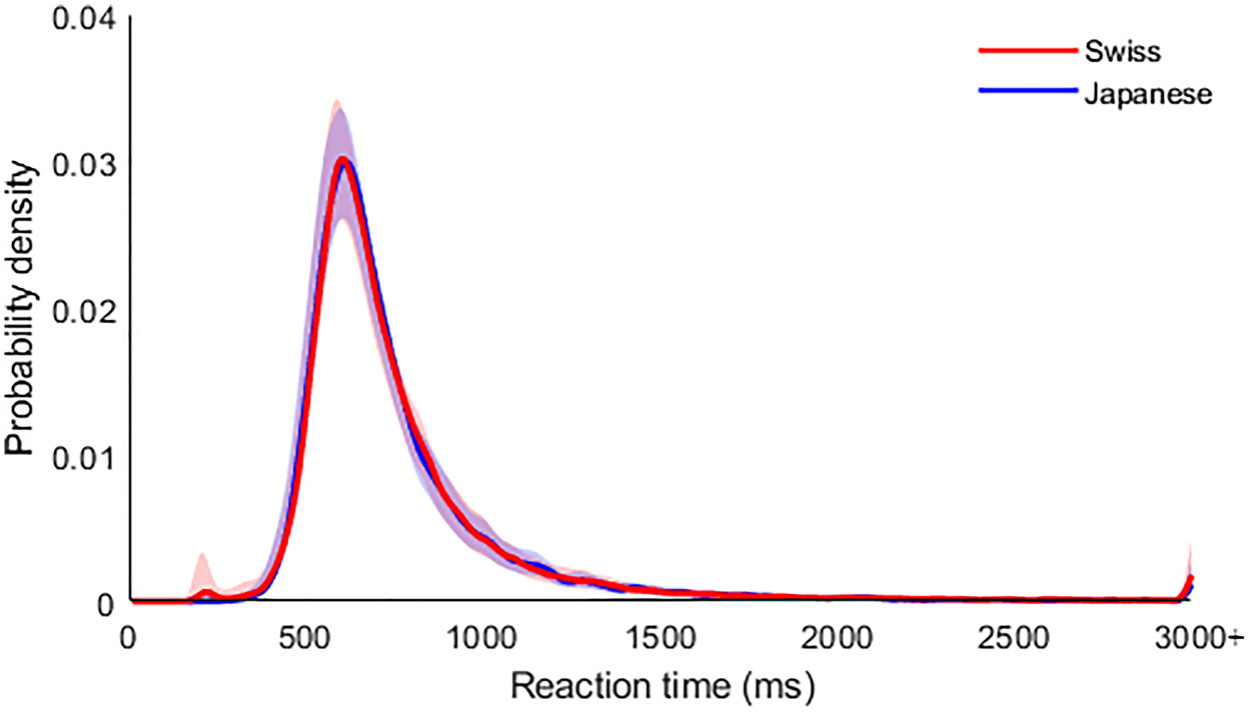
Correct reaction times for both Japanese and Swiss participants exhibited a comparable distribution, with 1.7% of trials exceeding the 2000 ms reaction time (RT) inclusion threshold for analysis. Data has been smoothed with a Gaussian-weighted moving average, and shaded areas represent 95% bootstrapped confidence intervals.

All participant datasets were assessed for inclusion/exclusion before statistical analysis. Trials with response times shorter than 100 ms and longer than 2000 ms were excluded from the datasets (0% and 1.7%, respectively, see Figure 2 for correct reaction time distributions prior to the trial exclusion). Participants with lower than 75% overall accuracy in the full-structure 100% coherence conditions were rejected (3% of participants in the Swiss group and 0% in the Japanese group). Similarly, participants that exhibited greater than 75% accuracy in either stimulus race conditions at the 0% coherence level were also rejected to exclude participants with biased response patterns when no face structure was discernible (35% in the Swiss group and 28% in the Japanese group). These processes also rejected participants who were not engaged in the task and made single-key responses (5% in the Swiss group and 10% in the Japanese group). Overall, these inclusion criteria excluded 22 datasets from each of the Swiss and Japanese participant samples, leaving 38 and 46 included datasets, respectively. It is likely that the less-controlled experimental context of the online study led to the high rejection rate, a potential methodological concern for such studies.

Condition means were calculated for each participant for accuracy (correct responses divided by accepted trials) and reaction time (RT, of correct trials). Accuracy and RT data were separately subjected to Bayes Factor ANOVA analyses for each of the 21 coherence levels comparing the effect of stimulus race, observer race, and the interaction between stimulus and observer race. The subject was treated as a random factor, and Bayes Factors were calculated across matched models (models with an interaction effect are compared with models of the same predictors yet excluding the interaction effect—see, Keysers et al., 2020). In addition to the Bayes Factor ANOVA, individual Bayes Factor analyses were conducted in the Swiss and Japanese observer data to provide additional insight into their respective behavioral profiles. Bayes factor thresholds representing sufficient evidence to denote the presence of an effect (difference in means) were taken as *BF*_10_ = > 3 and at *BF*_10 <_ = 0.33 for evidence supporting the null hypothesis. Evidence for the null hypothesis is presented in the figures containing Bayes factor analyses but is not explicitly discussed for brevity. Statistical analyses were conducted in RStudio (2021.09.2, 2020) using the BayesFactor package (0.9.12−4.3, Morey et al., 2018) and BayestestR (0.11.5, Makowski et al., 2019).

## Results

### ORCA Reaction Time

**Figure 3.**
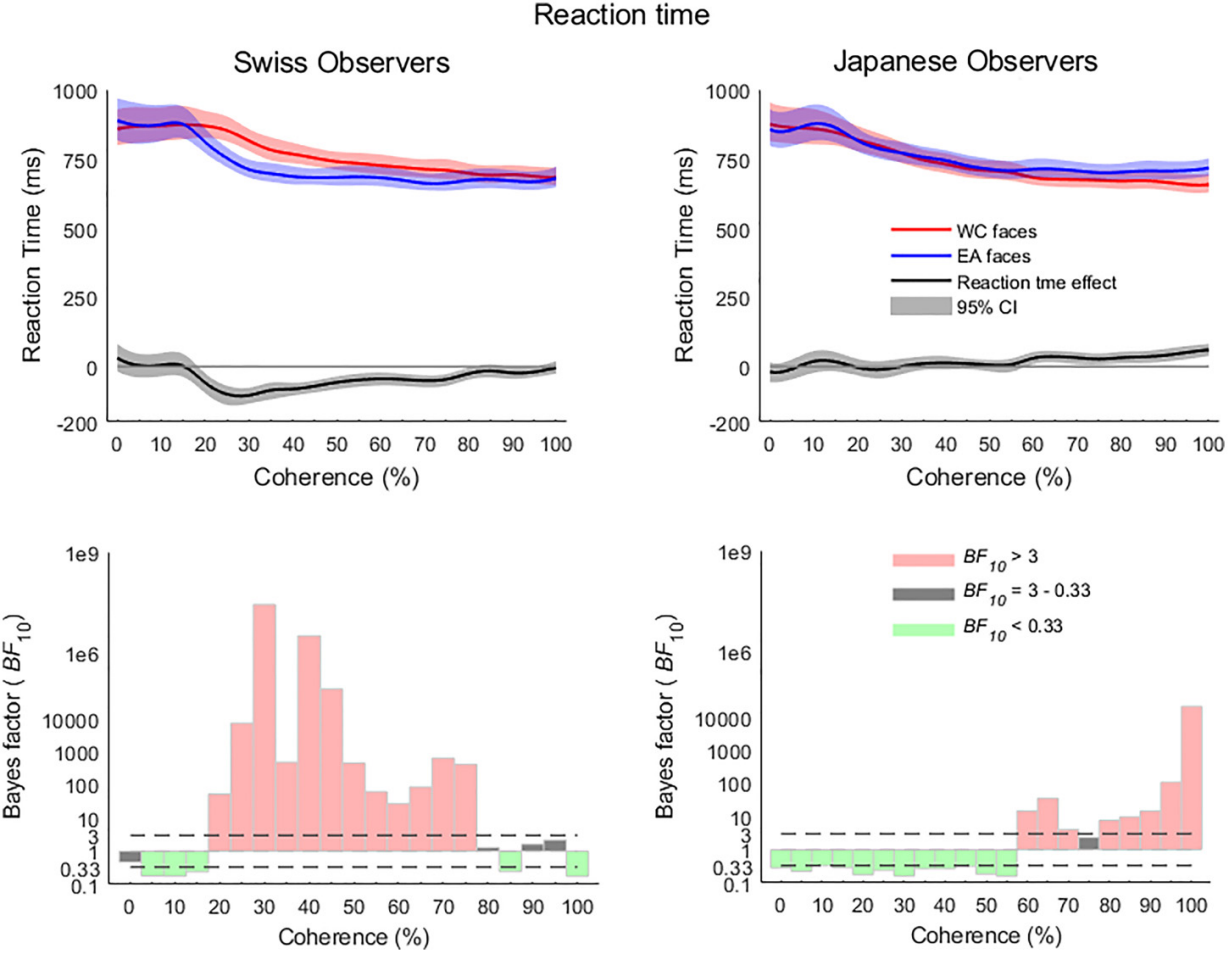
The reaction time profiles of the Swiss and Japanese observer groups exhibited a similar pattern to the accuracy data, with the exception of the 20% effect in the Japanese group. Reaction time profiles have been smoothed with a Gaussian-weighted filter and spline-interpolated for visual depictions. Shaded areas represent bootstrapped 95% confidence intervals.

The analysis of the Swiss observer group RT showed patterns of an ORCA RT effect from the 20% coherence level through to 75%, peaking at the 30% coherence level (*BF*_10_ = 2.62 × 10^7^). The Japanese group, however, exhibited clear evidence of an other-race categorization RT effect in the higher coherence levels from 60% through to 100% (peak effect at 100%, *BF*_10_ = 2.05 × 10^4^), excepting at 75% (see Figure 3).

**Figure 4.**
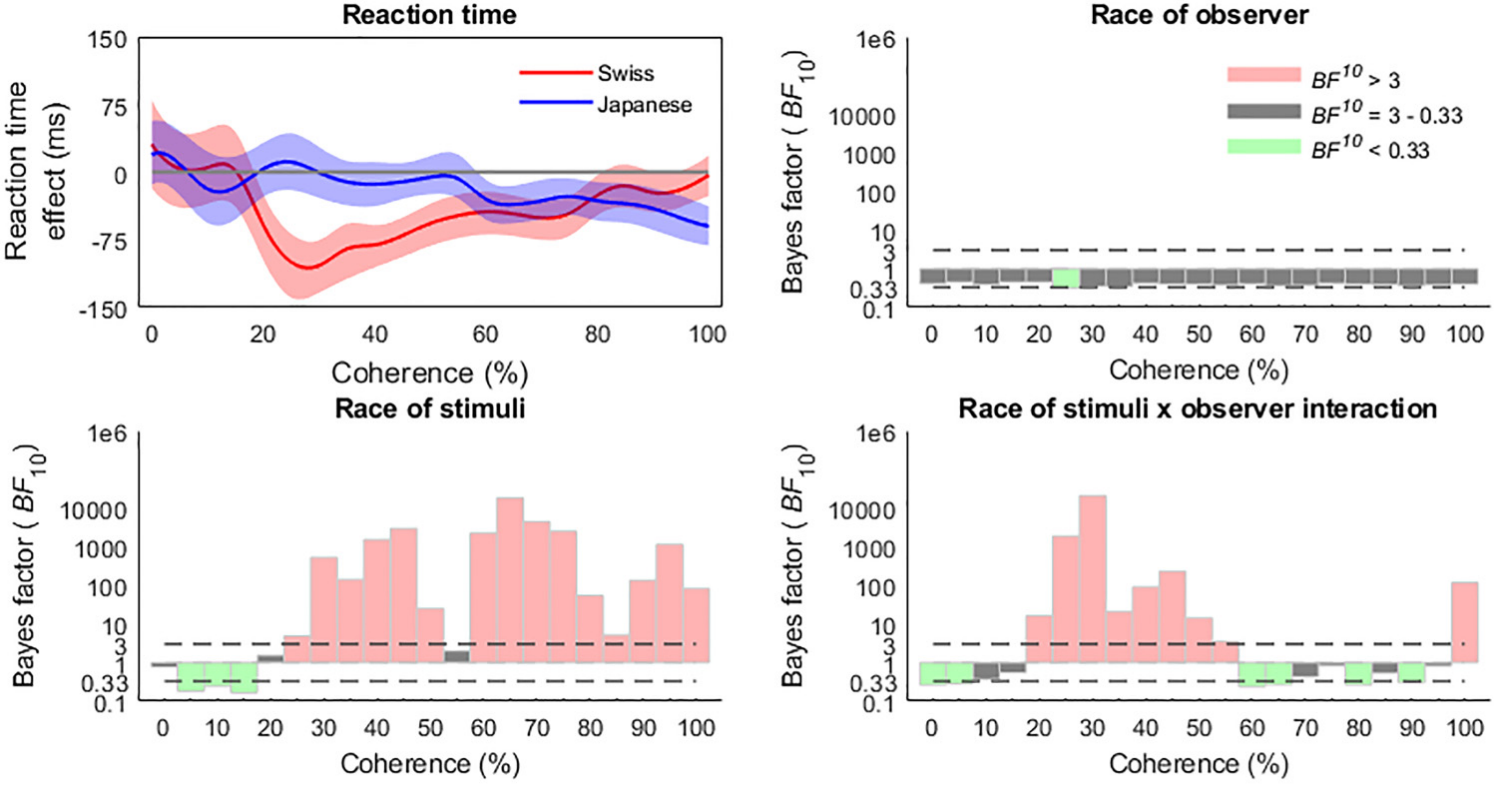
The Bayes Factor ANOVA for reaction time revealed evidence for an effect of race of stimuli across most of the coherence range starting from 25%, however, the interaction between stimulus and observer race showed that the low coherence level effects were due to the Swiss group while the high coherence level effect was due to the Japanese group. The reaction time effect is calculated as the other race minus same race reaction times and shaded areas represent bootstrapped 95% confidence intervals.

The Bayes Factor ANOVA for RT revealed evidence of an effect of the race of the stimuli from 25% through to 100% (peak 65%, *BF*_10_ = 1.83 × 10^4^), except at 55%. This general effect of stimulus race was moderated by the influence of observer race, with evidence of this interaction arising at 20% and continuing through to 55% (peak 30%, *BF*_10_ = 2.1 × 10^4^) and again at 100% coherence (*BF*_10_ = 117.87). The nature of the interaction is further illustrated in Figure 4, where the evidence for an effect of stimulus race in the lower coherence levels in the Swiss group contrasts with the evidence for the null hypothesis at those low coherence levels in the Japanese group, whereupon the pattern reverses in the high coherence levels.

### ORCA Accuracy

**Figure 5.**
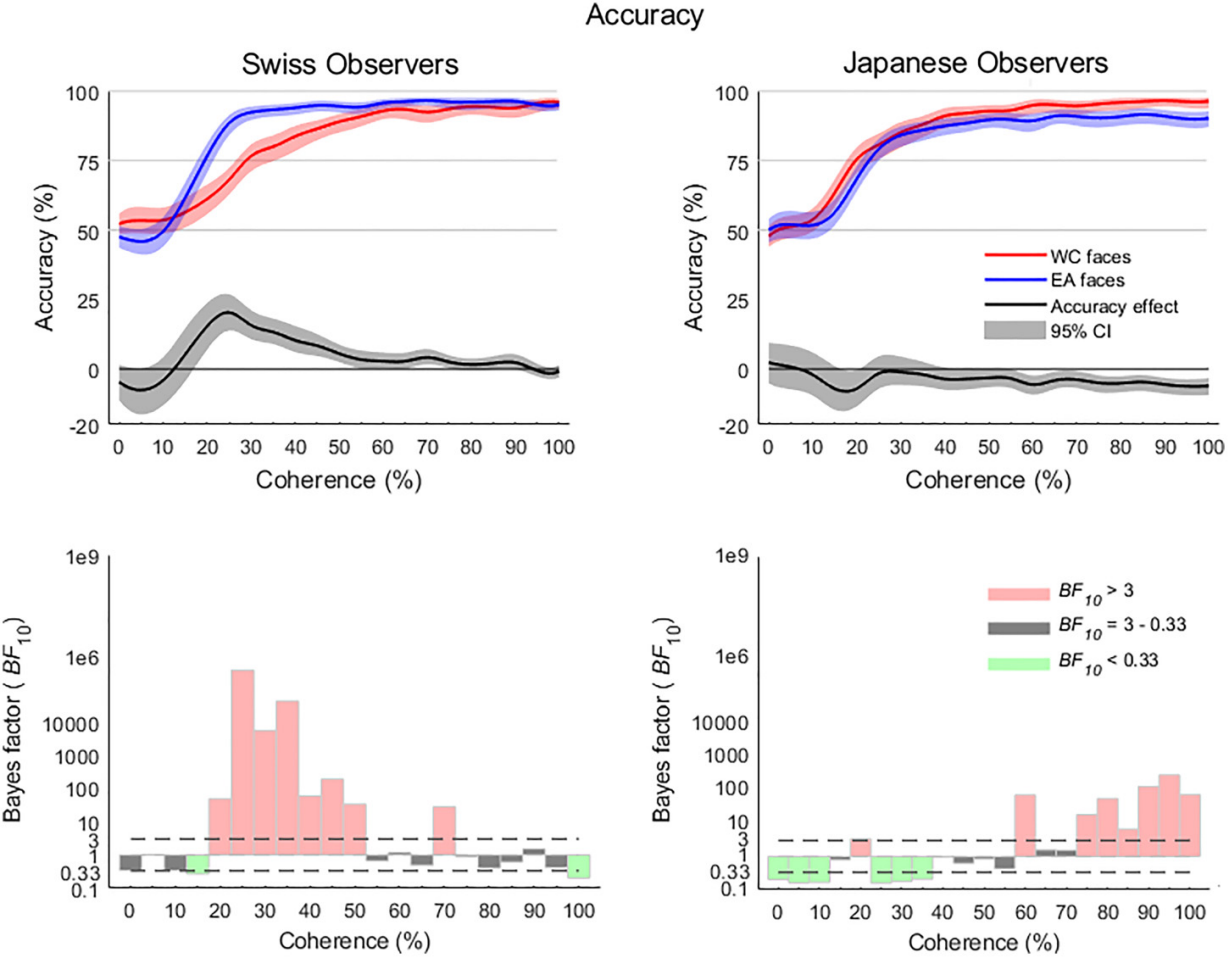
Accuracy at each level of coherence for the Swiss and Japanese observers revealed differing patterns, where the Swiss participants exhibited clear evidence of an Other Race Categorization Advantage (ORCA) in the low coherence levels while the majority of the effect for the Japanese participants was in the higher coherence levels. Accuracy profiles have been smoothed with a Gaussian-weighted filter and spline-interpolated for visual depictions Shaded areas represent bootstrapped 95% confidence intervals.

Analyzed separately to investigate the accuracy profiles of the two observer groups, both Swiss and Japanese participants exhibited chance (∼50%) level accuracy at very low coherence levels (see Figure 5), however at 20% coherence both groups showed very strong and moderate evidence that other race faces were categorized more accurately than same race faces (Swiss observers mean EA = 77.2%, WC = 60.9%, *BF*_10_ = 48.29, Japanese observers mean EA = 68.9%, WC = 77.5%, *BF*_10_ = 3.39). The accuracy advantage for other race faces continued in the Swiss group from 20% through to 50% and transiently at 70% coherence, peaking at 25% coherence (mean EA = 90.3%, WC = 67.4%, *BF*_10_ = 3.45 × 10^5^). The Japanese observers, however, did not exhibit a further other-race categorization advantage in the lower coherence levels, which instead manifested again in the higher coherence levels, transiently at 60% (mean EA = 88.6%, WC = 95.5%, *BF*_10_ = 70.78) and then sustained from 70% through to 100% with a peak at 95% coherence(mean EA = 89.7%, WC = 96%, *BF*_10_ = 280.13).

**Figure 6.**
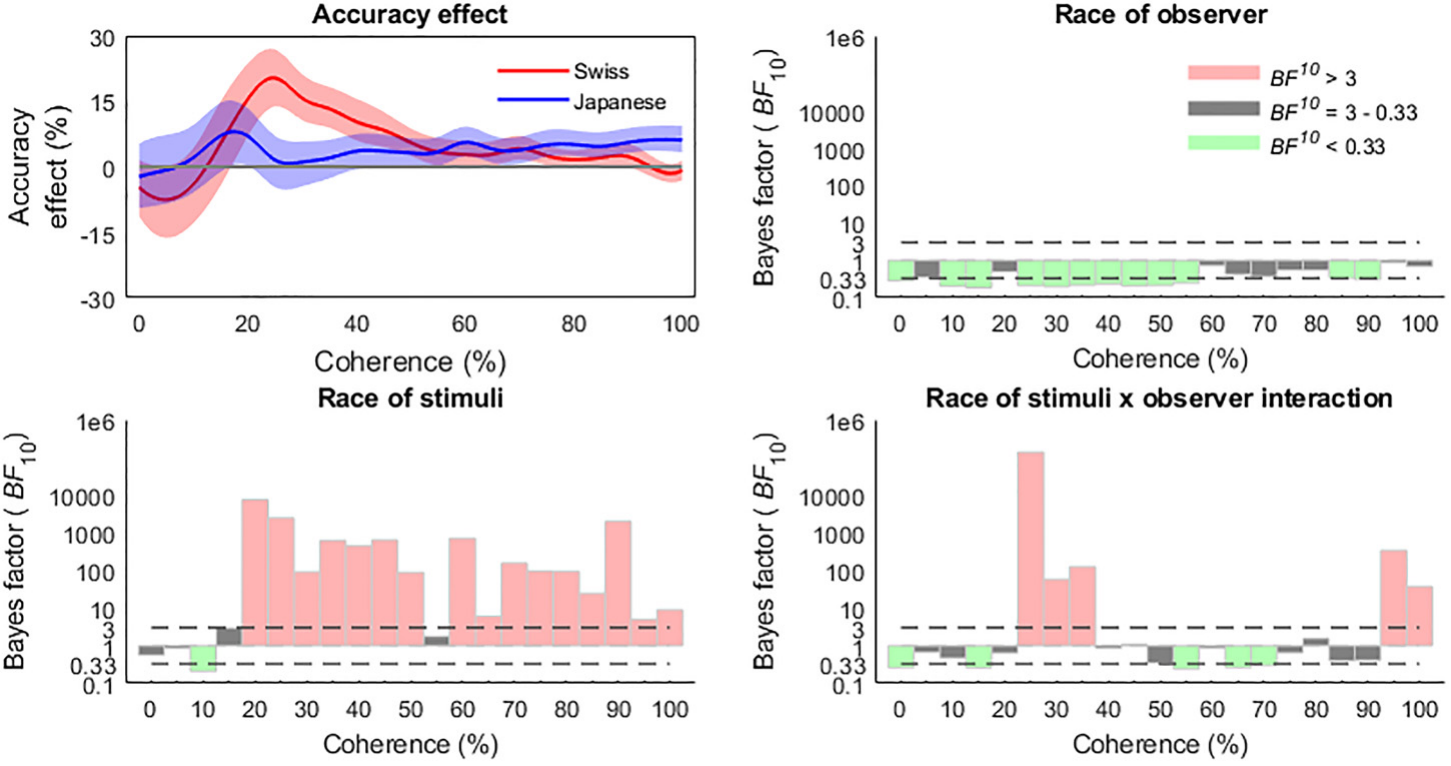
Bayes factor ANOVA results revealed an other-race categorization advantage for accuracy from 20% and enduring through most of the higher levels of coherence, although this effect was moderated by different accuracy profiles for the Swiss and Japanese observers. Accuracy effect is calculated as other race minus same race accuracies and shaded areas represent bootstrapped 95% confidence intervals.

The Bayes Factor ANOVA analysis comparing the effects of stimulus race, observer race, and stimulus × observer race interaction suggested that the race of the observer itself did not influence the accuracy of race categorization, but that the effects at various levels of phase coherence were moderated by an interaction between stimulus and observer race. The main effect of stimulus race arose at 20% coherence (also representing the peak of the effect, *BF*_10_ = 7.6 × 10^3^), and was present throughout the range of coherence levels, excepting 55% (see Figure 6). However, the stimulus × observer race interaction effects clarify these patterns, revealing an enhanced other-race categorization effect for the Swiss group in the lower coherence levels from 25% to 35% (peaking at 25%, *BF*_10_ = 1.37 × 10^5^), while the Japanese group exhibited enhanced sensitivity to other races in the higher 95−100% coherence levels (peaking at 95%, *BF*_10_ = 336.51). Of note, there was no evidence of a race of stimulus × observer race interaction in the 20% coherence levels.

## Discussion

The analyses of race categorization reaction times revealed contrasting patterns for the Swiss and Japanese observer groups, with the former exhibiting robust evidence of an ORCA when the image structures had been greatly degraded (from 20% through to 50% phase coherence). The Japanese observer group, however, only exhibited a reliable ORCA in reaction time when there was more structure in the face images, from 60% through (mostly) to the conventional 100% phase signal. The Bayes Factor ANOVA confirmed this pattern, with a clear pattern of stimulus race by observer race interactions in the lower coherence levels and at the 100% level (Figure 4). The pattern for the Japanese group thus aligned with previous results finding ORCA reaction time effects when using clear, full-structure (unscrambled) face images as stimuli (Caldara et al., 2004; Feng et al., 2011; Ge et al., 2009; Levin, 1996, 2000; Valentine & Endo, 1992; Zhao & Bentin, 2008, 2011). The reaction time patterns of the Swiss group, however, did not align with these past results using 100% phase signals, even when cross-cultural participant samples were used. This might be due to very repetitive nature of the task. It is worth noting that, the reaction time profiles did not show evidence for a race of observer effect for reaction time at any of the coherence levels. Rather, the ORCA observed at different coherence levels suggests that the visual race signals extracted and used in race categorization tasks do not follow a universal trajectory as images are systematically degraded of structure, but that the race/culture of the subject plays a strong role in what visual information is extracted and used. While in our study we specifically manipulated the amount of visual structure present in the face images, previous studies have found evidence that Caucasian and Asian observers often use differing levels of high and low spatial frequencies in face processing tasks, specifically that Asian participants typically tend to rely more heavily on low-spatial frequencies, while Caucasian participants make more relative use of high spatial frequencies (Blais et al., 2008; Caldara et al., 2010; Estéphan et al., 2018; Miellet et al., 2010; Tardif et al., 2017; for a review Caldara, 2017). While the phase-scrambling procedure randomly misplaces the location of differing levels of image structures and thereby retains the same profile of spatial frequency information across the images, it necessarily decreases or completely erodes the spatial frequencies able to be extracted to form the structural patterns of the forms in the image—in our case that of the faces. It is possible then that the patterns we observe in the current study are related to those found for spatial frequency in cross-cultural comparisons, specifically the emphasis on higher spatial frequencies for Asian participants and the lower frequencies for Caucasians. Here it should be reiterated that the phase-scrambling technique used to systematically degrade the visual structure of the faces was applied equally to the EA and WC stimuli and that they were balanced for brightness, contrast, and spatial frequency content at each level of phase-coherence. The effects observed are thus unlikely to be due to low-level visual differences introduced by the phase-scrambling technique, but rather the different ways in which the Swiss and Japanese participants extracted race-relevant visual signals from them.

As the effects observed in the current study related to the perception of the race of the stimuli used, it is relevant to consider the possibility that the Chinese identities used as the EA stimuli may have been processed as an other-race or an out-group by the Japanese participants in the current study, though different by degrees to the WC Swiss faces used. A recent study utilizing the very same EA and WC stimuli in both Swiss and Japanese participant samples observed a robust ORCA, exhibiting a cross-cultural interaction when tasked with categorizing the face by race (de Lissa et al., 2021). We can thus rule out the possibility that our current results rely on a specific difference in how the Swiss and Japanese groups perceived in/out group faces.

**Figure 7.**
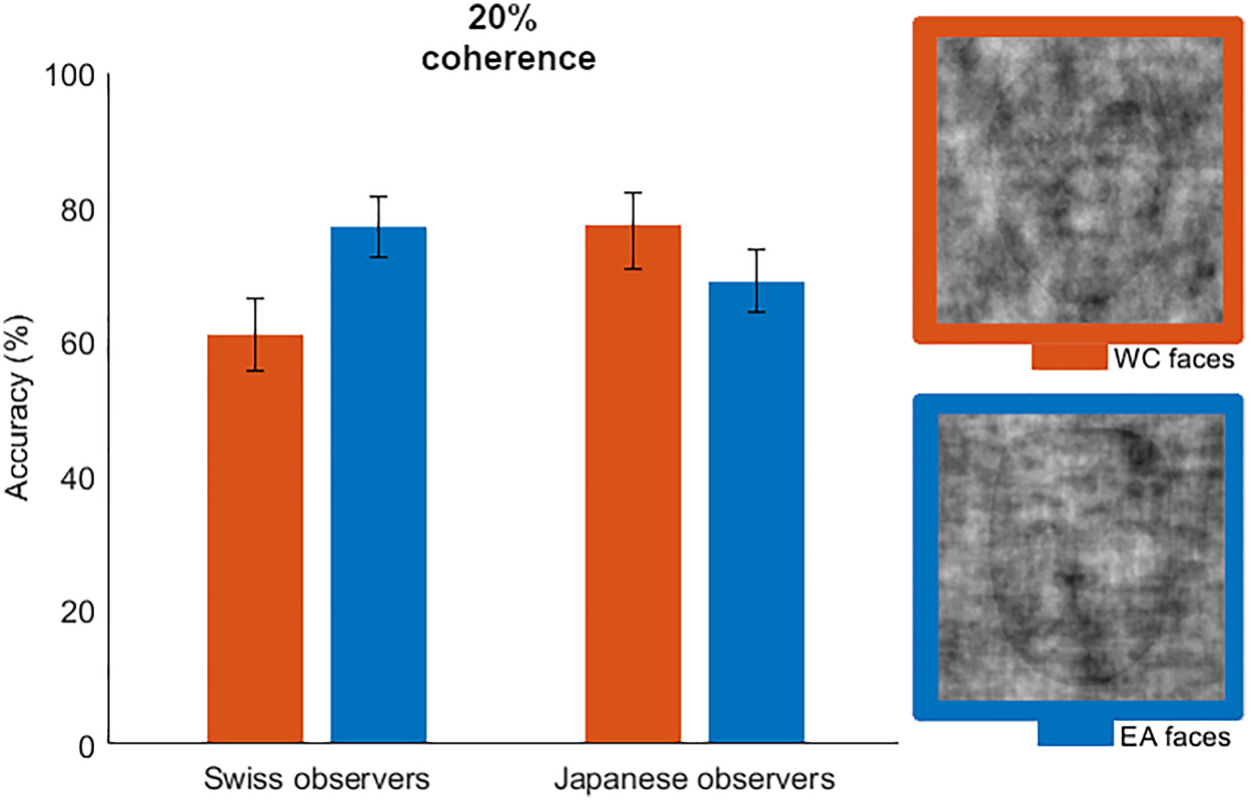
Both observer groups exhibited an Other Race Categorization Advantage (ORCA) in accuracy at the 20% coherence level, with no evidence of a group or interaction effect. The example stimuli provide an indication of how little structure was sufficient to elicit this effect for each group and each stimulus, respectively.

It is also important to note that we observed clear evidence of an ORCA in both reaction time and in accuracy. While most studies investigating the ORCA have observed mostly reaction time effects (Blais et al., 2008; Caldara et al., 2004; Ge et al., 2009; Liu et al., 2015; Valentine & Endo, 1992; Zhao & Bentin, 2008, 2011), the effects we observed in reaction time were mostly also present in the accuracy analyses. While less robust than reaction time, ORCA accuracy has been observed in a few previous studies (de Lissa et al., 2021; Feng et al., 2011). In the current study, it was hypothesized that if people exhibit enhanced sensitivity to other race visual signals then systematically degrading the visual quality of faces may lead to a point where other race signals remain perceptible while same race signals are not. Apart from the cultural differences we observed in ORCA accuracy across different levels of phase coherence (visual structure), both observer groups exhibited clear evidence of an accuracy ORCA at the 20% coherence level (see Figure 7). Although the Japanese group exhibited less evidence than the Swiss group (*BF*_10_ = 3.39 compared with *BF*_10_ = 48.29, respectively), the overall effect of stimulus race was clear in the accuracy of Bayes Factor ANOVA at 20%, and the stimulus by observer race interaction only became evident at the 25% coherence level. At the 20% level of coherence, the Swiss and Japanese groups attained an average accuracy of ∼77% for other race faces, while the Japanese group was on average better at categorizing same race races than the Swiss group (69% and 61%) although this was not evident in the Bayes Factor results. The clear pattern of evidence for an ORCA for accuracy, in our view, reflects an enhanced sensitivity for the visual race signals of other race faces, having reached a level of degraded visual input where the different levels of activation threshold for the same and other race faces led to corresponding differences in the ability to perceive the relative race signals. At this point, it is important to reiterate that the visual stimuli themselves were balanced such that even though the same and other race faces were significantly degraded in visual structure (see Figure 7), the phase scrambling process did not benefit one stimulus over another. In addition to this, the stimuli were themselves counterbalanced in terms of what constituted the “same” and “other” race for the two observer groups. We, therefore, suggest that the accuracy effect manifesting in response to the heavily degraded stimuli (20% coherence) reflects a universal sensitivity to other race faces, and may be independent of the observer group ORCA interactions occurring across the other coherence levels.

Such a universal effect is in line with the multidimensional face space model advanced by Valentine (1991), Valentine and Endo (1992), decreased familiarity with other races leads to them being stored in as a more dense cluster of exemplars (see also Caldara & Abdi, 2006), where the density of the array of exemplars for other race faces would most likely be influenced most strongly by a common salient visual feature shared across other-race face exemplars. Accordingly, our results are in line with those of Levin (1996, 2000), although we do not advance a position on what “features” contain the race signals, nor do we have a clear view of how such signals might be emphasized when performing implicit social categorization tasks. Here, it is worth pointing out an uncertainty in the generalizability of the patterns we have observed in our study in tasks not requiring explicit race categorization. Not only that, the difference in the accuracy and reaction times between our Swiss and Japanese observers may involve not only implicit differences in how race is extracted and processed across different levels of visual degradation, but that differences in the way that the task may be approached might also need to be considered. Such a consideration is particularly relevant when considering how the perception of “threat” might interact with the perception of race or the specific tasks/instructions given to participants. We did not control for the apparent perception of threat, dominance, attractiveness, etc., instead utilizing face stimuli displaying a neutral facial expression of emotion that had been found to elicit a cross-cultural ORCA interaction in a previous study (de Lissa et al., 2021). However, these dimensions may significantly interact or overlap with the categorization of race by facilitating fast responses, particularly in paradigms involving short presentation times where the extraction of visual features may be impacted due to hierarchical chains of processing from course to fine analysis of face structures/features (Petras et al., 2019).

Our results also have implications for future studies and the general consideration of how visual degradation of images may modulate the strength of other race effects in real life. While it is valid to assume that the visually clear images used in previous studies to investigate the ORE and ORCA are good representations of face images we might commonly encounter in life, we are also often presented with photographs or videos where the image quality is significantly reduced. Similarly, a dark environment or ones involving other types of visual degradation might be more likely to lead to an enhanced perception of race in other race faces relative to same race faces, to the point where race might only be extracted from the former. This has the potential to impact the reporting of descriptions of possible suspects in law enforcement contexts, leading to an over-representation of reports specifying race when other (less familiar) race is involved. Further, our results raise questions about how the brain responds to faces of different races when visual quality has degraded to an extent where underlying sensitivities to the same and other race signals may lead to either the presence or absence of activation. At such levels of visual degradation, it is possible that the neural indices of race processing and perception are at their most clear when comparing the response to the same and other race faces. Accordingly, such a paradigm might be well-suited to determine the timing of when the race is processed in the brain (e.g., Vizioli et al., 2010a, 2010b), as the presence versus the absence of activation may provide a clearer lens through which to see such an effect, whereas such an effect might not be as apparent when activation of the race perception processes occur reliably in each trial in response to full-structure face images.

### Conclusions

In one of the earliest scientific investigations of the perception of other race faces, Feingold (1914) posited that our insensitivity to visual qualities signifying our own race is a “matter of habit,” in that we do not find such signals diagnostic or useful in the tasks we perform when viewing faces where there is not much racial diversity. When introduced to new physiognomic patterns characteristic of other races, such visual signals provide a salient marker of difference. It is only through repeated encounters that the salience of such markers becomes less diagnostic, as we are met with the need to individuate members of other races more often. Our findings point to this enhanced sensitivity to visual race signals in other race faces, which in the current study manifested when the visual quality of the face images was degraded to a critical threshold. However, the additional findings of the two observer groups exhibiting modulations of an ORCA in accuracy and reaction time suggest that the additional consideration of observer-group social/cultural aspects when interpreting the relative presence or absence of such effects using traditional broad-band and full-structure faces.
